# Case Report: Narcolepsy associated with anti-Ma2 antibody positivity

**DOI:** 10.3389/fimmu.2026.1942979

**Published:** 2026-10-05

**Authors:** Yefan Lv, Hui Lv, Yike Huang, Xiaoyan Long, Jinxia Zhou

**Affiliations:** 1 Department of Neurology, Xiangya Hospital, Central South University, Changsha, Hunan, China; 2 Department of Neurology, Fifth Affiliated Hospital of Sun Yat-Sen University, Zhuhai, Guangdong, China

**Keywords:** cataplexy, narcolepsy, immunotherapy, anti‑Ma2 antibody, anti‑Yo antibody

## Abstract

**Background:**

Several intraneuronal antibodies have been identified in association with paraneoplastic encephalitis, including anti-Ma and anti-Yo antibodies. There is a pure case of narcolepsy associated with anti-Ma2 antibody positivity with concomitant anti-Yo antibody.

**Case presentation:**

We report a 42-year-old male patient who presented with recurrent cataplexy as the primary and dominant symptom without typical hyperphagia in the early stages of narcolepsy associated with anti-Ma2 antibody positivity. Despite extensive screening, no underlying malignancy was detected over a 3-year disease duration. The patient exhibited significant clinical improvement following immunotherapy at the beginning.

**Conclusions:**

This is a rare report of an isolated clinical manifestation of narcolepsy associated with anti-Ma2 antibody positivity with concomitant anti-Yo antibody. This case reminds clinicians to notice that cataplexy could be the only early symptom of narcolepsy associated with anti-Ma2 antibody positivity with concomitant anti-Yo antibody, which can easily lead to misdiagnoses. Autoimmune mechanisms may play an important role in the pathogenesis of narcolepsy associated with anti-Ma2 antibody positivity, and prompt immunotherapy offers a promising approach for its management.

## Introduction

Several intraneuronal antibodies have been identified in association with paraneoplastic encephalitis, including anti-Ma2 antibodies (Ma2-Abs) and anti-Yo antibodies (1). Ma2 is a common antigen of the Ma family, and its antibodies are highly targeted throughout the brain, particularly in the limbic structures, brainstem, hypothalamus, and cerebellar nuclei (2). Cases of anti-Ma2 encephalitis commonly present with limbic, brainstem, and diencephalic encephalitis, often in isolation or combination (3). Clinical symptoms and signs are conditional on the location and severity of encephalitis. The accompanying narcolepsy represents a rare and atypical manifestation indeed. In a previous study involving 38 patients, 32% of them exhibited early and obvious excessive daytime sleepiness (EDS). Among patients with limbic system or diencephalic dysfunction, this proportion reached 41%. At the same time, the patients had low or undetectable levels of hypocretin in their cerebrospinal fluid (CSF) (3). Therefore, it was speculated that EDS might be caused by hypothalamic dysfunction (3). At the same time, cataplexy was occasionally reported (4, 5). These cases are often associated with tumors, such as testicular or lung malignancies in male individuals (3). The anti-Yo antibody targets a protein highly concentrated in the cerebellum and is primarily found in women with paraneoplastic cerebellar degeneration associated with breast or ovarian cancer. Few cases have been reported in male patients with nasopharyngeal carcinoma who tested positive for Anti-Yo, which led to limbic encephalitis (6). To the best of our knowledge, only a few cases of a narcolepsy-like syndrome associated with non-paraneoplastic anti-Ma encephalitis have been previously reported. Here, we report a case of a patient with narcolepsy associated with anti-Ma2 antibody positivity with concomitant anti-Yo positivity, who presented with recurrent cataplexy as the primary and dominant symptom, showing no evidence of tumor in three years’ follow-up and demonstrating a good response to immunotherapy at the beginning.

## Case presentation

A 42-year-old male was admitted for recurrent episodes of limb weakness and falls for 2 years. Initially, the patient experienced sudden loss of muscle tone and falls lasting 3–5 seconds while ambulating, occurring 1 to 2 times daily. Electroencephalogram monitoring was observed for 24 h and was normal, during which the patient had no paroxysmal events identified. Neurological examination was unremarkable. Epilepsy was suspected, and magnesium valproate was prescribed. The antiepileptic treatment was proven ineffective over two months and discontinued. Later, he had hyperphagia with a 12kg weight gain over six months and developed daytime hypersomnolence with an Epworth Sleepiness Scale (ESS) score of 14.

Polysomnography revealed a total sleep time of 330 minutes, sleep efficiency of 56.3%, sleep latency of 0 minutes, and an apnea-hypopnea index of 75.6/h, indicative of severe sleep apnea syndrome (**Figure 1A**). During rapid eye movement (REM) sleep, increased tonic and phasic submental electromyographic activity was observed associated with vocalization and limb movements. The patient was considered to have a REM sleep behavior disorder (RBD). A 5-nap Multiple Sleep Latency Test (MSLT) disclosed a short mean sleep latency of 3 minutes and 6 seconds, with the presence of 3 sleep-onset REM periods and a mean REM-onset latency of 1 minute and 20 seconds (**Figure 1B**), and complete loss of REM sleep atonia. The patient exhibited good compliance with continuous positive airway pressure (CPAP) therapy for half a year. CPAP substantially lowered the apnea-hypopnea index, but daytime sleepiness was not alleviated. The repeated polysomnography and MSLT were acquired off CPAP on the recording night. Repeated polysomnography indicated a total sleep time of 466 minutes, sleep efficiency of 82.6%, sleep latency of 0.5 minutes, and an apnea-hypopnea index of 80/h without CPAP. Meanwhile, we found that REM sleep accounted for 8.8% of total sleep time, accompanied by dream-enactment behavior and increased submental electromyographic activity. A repeated MSLT remained positive, which demonstrated a short mean sleep latency of 1 minute and 30 seconds, with two sleep-onset REM periods and a mean REM-onset latency of 0 minutes (**Figure 1C**). Brain MRI findings were unremarkable. The serum levels of tumor markers were within normal ranges, such as α-fetoprotein, β-human chorionic gonadotropin, and lactate dehydrogenase. Inflammatory factors in the serum were abnormal. Specifically, the level of tumor necrosis factor-α was 14.1 pg/ml, and the level of interleukin-1β was 8.06 pg/ml. Cerebrospinal fluid (CSF) analysis showed elevated protein levels of 0.68 g/L and a normal cell count. Meanwhile, CSF immunoglobulin G level was elevated at 0.09 g/L. The CSF hypocretin-1 level was 5.9 pg/mL (normal >200 pg/mL). Human leukocyte antigen (HLA) typing was positive for DQB1*06:02 and DQB1*03:01. The patient was diagnosed with narcolepsy type 1 (NT1) and treated with sertraline hydrochloride and methylphenidate. While cataplexy initially improved, it worsened significantly three months later, occurring 2–10 times daily. Subsequently, analysis for anti-Ma2 and anti-Yo antibodies was performed using immunoblotting of recombinant neuronal antigens (EUROIMMUN Neuronal Antigens Profile, Lübeck, Germany). The testing was performed at the Department of Clinical Laboratory, Xiangya Hospital. According to the manufacturer’s criteria, EUROLineScan chromaticity values of 0-5 were defined as negative, 6-10 as borderline, 11-50 as positive, and >50 as strongly positive. For this case, the semi-quantitative chromaticity values of anti-Ma2 were 24 and anti-Yo were 16 in the serum, and anti-Ma2 were 3 and anti-Yo was 1 in the CSF, with the testing date on January 4, 2024. This assay showed 89 % (overseas) and 95.2 % (Chinese-cohort) sensitivity for anti-Ma2, 100 % (overseas) and 97.1 % (Chinese-cohort) sensitivity for anti-Yo, with 100 % overall specificity (manufacturer-provided validation data, EUROIMMUN EUROLINE Neuronal Antigens Profile (IgG) package insert, catalogue No. DL 1111-1601-2-G). The laboratory tests revealed positive anti- Ma2 and anti-Yo antibodies in the blood, though CSF tests were negative for these antibodies. Comprehensive oncological screening*s* were negative, including a whole-body ¹^8^F-fluorodeoxyglucose-positron emission tomography-computed tomography, thorax computed tomography scan, abdomen echography, and testis echography. A diagnosis of NT1 associated with anti-Ma2 antibody positivity was established. The patient underwent two courses of plasma exchanges. The first session removed 1300 mL of plasma, and the second session removed 1278 mL of plasma, with an interval of five days between each session. Immediately following that, intravenous immunoglobulin at a dose of 0.4 g/kg/day for five consecutive days was administered at a two-day interval after completion of the second plasma-exchange course, resulting in significant improvement in cataplexy as reported by the patient. He declined to retest the hypothalamic orexin levels and to undergo further second-line immunotherapy due to economic factors. After immunotherapy, partial improvement in cataplexy was observed before the initiation of symptomatic medications. The patient was administered methylphenidate and venlafaxine for residual sleep-related symptoms. Follow-up at 12 months showed significant clinical improvement, with an ESS score of 6 and only two cataplectic attacks reported over the entire year. Repeated blood tests for autoantibodies, including anti-Ma2 and anti-Yo, were negative. At the 3-year follow-up, the patient’s cataplexy gradually became more frequent and severe. Repeated anti-Ma2 and anti-Yo tests were again positive, but repeated evaluations for underlying malignancies remained negative, including thorax computed tomography scan, thyroid ultrasonography, abdominal echography, penile ultrasonography, and testicular echography. Meanwhile, Brain MRI and tumor markers in serum still showed no abnormality. The patient rejected all immunotherapy due to economic factors. A graphical overview of the evolution of the patient’s clinical course, auxiliary tests, diagnosis, and treatment intervention measures is presented on a timeline (**Figure 2**).

**Figure 1:**
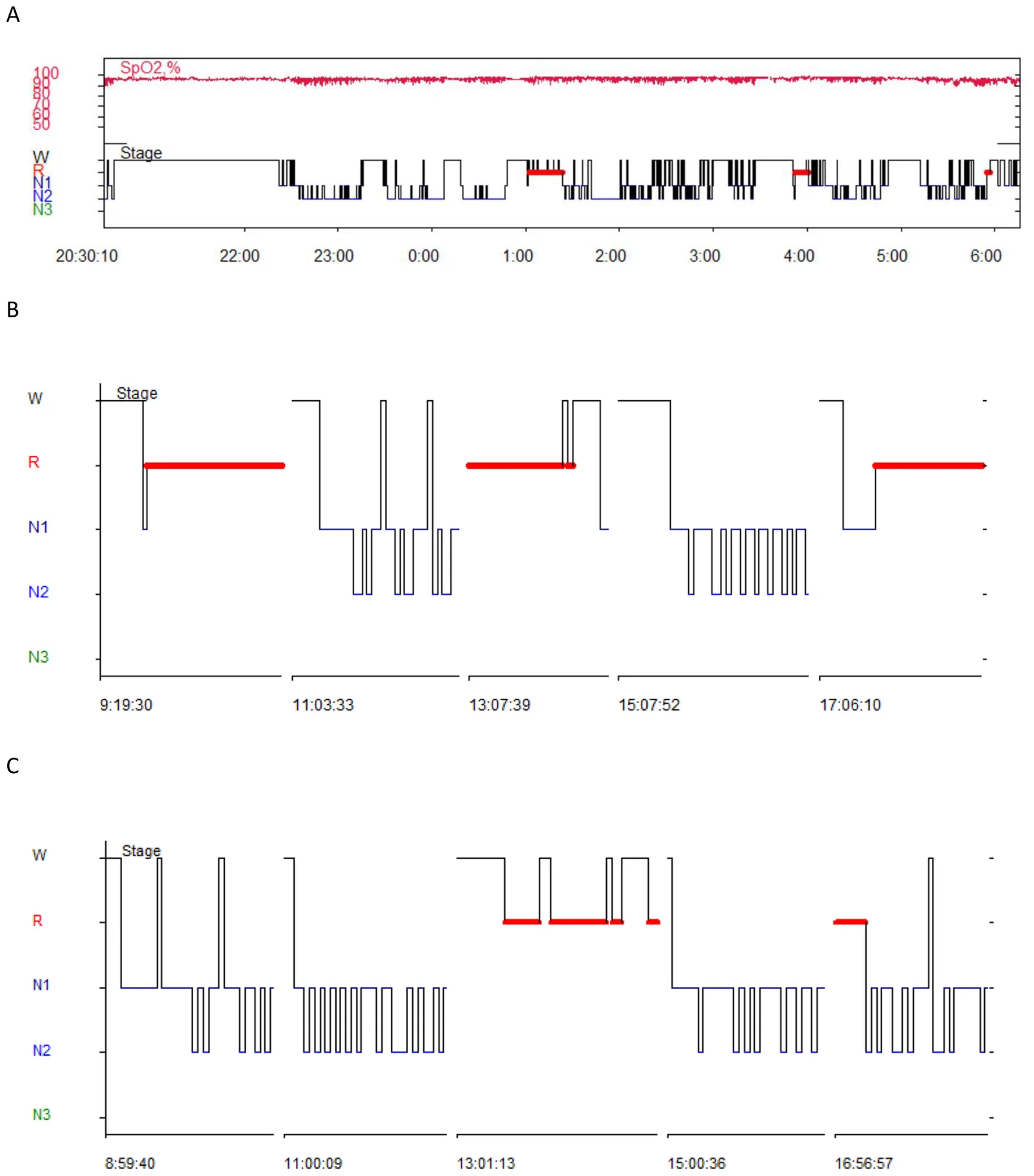
Graphs of hypnogram. **(A)** whole night sleep structure recorded by Polysomnography. **(B)** Multiple sleep latency test before continuous positive airway pressure treatment. It shows 3 sleep-onset REM periods out of 5 naps, with a short mean sleep latency of 3 minutes and 6 seconds and a mean REM-onset latency of 1 minute and 20 seconds. **(C)** Multiple Sleep Latency Test after continuous positive airway pressure treatment. It discloses 2 sleep-onset REM periods with a short mean sleep latency of 1 minute and 30 seconds and a mean REM-onset latency of 0 minutes.

**Figure 2:**
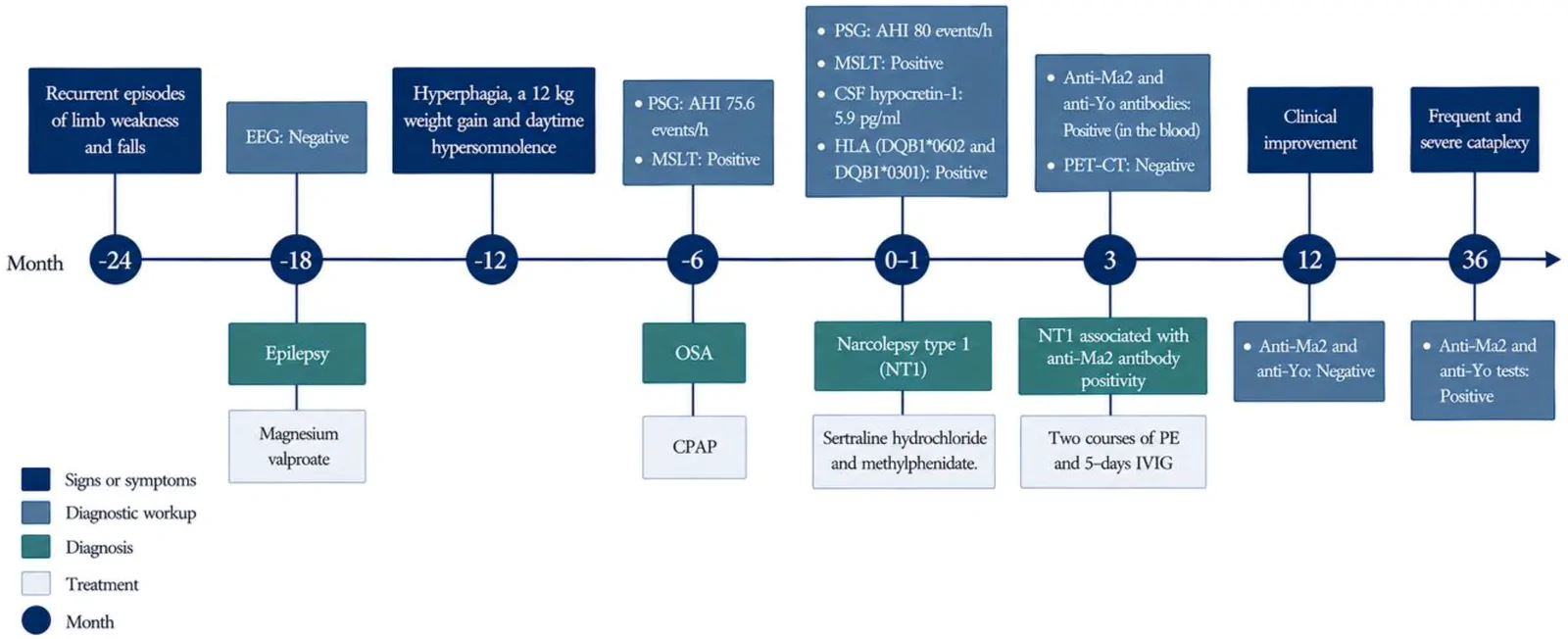
Clinical timeline of disease course and treatment. The timeline describes the clinical process, diagnosis, treatment and outcomes.

## Discussion and conclusions

According to the 2021 paraneoplastic neurologic syndromes-care criteria, anti-Ma2 represents a high-risk onconeural antibody frequently linked to underlying malignancy, which may cause diencephalic injury presenting as narcolepsy-cataplexy (7). A definite diagnosis of anti-Ma2-associated paraneoplastic encephalitis requires compatible tumor evidence (7). According to the latest research, anti-Ma2-related autoimmunity can be classified into two subtypes: idiopathic Ma2-Abs (I-Ma2) and paraneoplastic Ma2-Abs (PNS-Ma2) (8). The I-Ma2 subtype accounts for nearly one-third of affected patients, in whom no malignancy is detected with a minimum follow-up of at least 2 years after diagnosis, and it is typically characterized by an insidious onset and a predilection for monofocal nervous system involvement. Isolated serum antibody positivity with negative CSF findings from commercial line-blot assays needs orthogonal confirmatory testing to rule out false-positive results (7). In our patient, only serum anti-Ma2 was positive, while CSF antibody testing and oncological screening were unremarkable. Residual samples were exhausted for further validation. Hence, we describe it as narcolepsy associated with anti-Ma2 antibody positivity. Our patient presented with purely diencephalic symptoms, along with positive Ma2 antibodies and elevated inflammatory markers in the serum, as well as increased IgG protein in the CSF. Although the antibodies in CSF and MRI results were unremarkable, anti-Ma2-related diencephalic involvement may occur without typical MRI abnormalities (3). Notably, consensus guidelines emphasize higher diagnostic yield of CSF testing for neuronal surface antibodies, such as anti-NMDAR, anti-LGI1, anti-CASPR2, and anti-AMPAR (3, 7); yet simultaneous serum and CSF testing is still recommended for intracellular onconeural antibodies such as anti-Ma2. A previous report by Dalmau et al. have shown that few cases of anti-Ma2 encephalitis were accompanied by negative T2-weighted MRI scan results and/or anti-Ma2 antibodies in CSF (3). The novelty of our case lies in the presentation of narcolepsy-cataplexy in the setting of concurrent serum anti-Ma2 and anti-Yo antibody seropositivity. Cataplexy was the primary and dominant symptom in this case. Anti-Ma2-associated encephalitis is clinically characterized by upper brainstem, limbic system, hypothalamic, and cerebellar impairment. Excessive daytime sleepiness is a common feature in anti-Ma2-associated encephalitis, occurring in up to 32% of subjects, while cataplexy has been reported in less than 3% (9). In the current case, the patient presented with narcolepsy with cataplexy. The absence of hyperphagia in the early stages of this case led to initial misdiagnosis. The neurochemistry of cataplexy, originally focused on catecholamines and acetylcholine, now extends to hypocretin, which is believed to prevent cataplexy (10). Hypocretin is a neuropeptide of hypothalamic origin that promotes wakefulness and prevents cataplexy. Idiopathic NT1 is characterized by selective loss of hypocretin-producing neurons in the posterior hypothalamus. In the current case, a markedly low level of CSF hypocretin was also observed in anti-Ma2 and anti-Yo antibodies associated with narcolepsy. Dauvilliers et al. found that the neuropathology of a patient with anti-Ma-associated diencephalitis was especially marked by inflammation of the hypothalamus during the 4 months between diagnosis and death, with clinical presentations of oversleeping, cataplexy, and brainstem syndrome (5). The study revealed that a CD8^+^ inflammatory-mediated immune response specifically targets hypocretin-producing neurons in the hypothalamus in narcolepsy–cataplexy secondary to anti-Ma2-associated encephalitis (5).

The HLA DQB1*06:02 allele is closely associated with idiopathic NT1, present in over 90% of affected individuals, and increases the risk of developing NT1 by 200-fold. However, our case suggests that it can also be present in secondary narcolepsy, and we speculate (without direct supporting evidence) that it is likely to increase the immune attack on the hypothalamus in patients with Ma2-Abs. Previous studies have also found that KLHL11 antibody-related encephalitis was closely associated with HLA-DRB1*03:01 and HLA-DQB1*02:01. This type of encephalitis is also often accompanied by testicular cancer, where HLA class II promotes CD4^+^ T cell activation, which in turn activates CD8^+^ effector cells and B cells (11). Anti-Yo antibody encephalitis is characterized by paraneoplastic cerebellar degeneration associated with breast cancer, nasopharyngeal carcinoma, or ovarian cancer (1). However, our case found positive anti-Yo antibodies. Immune generalization represents one speculative explanation for this serological co-positivity; however, we could not confirm a direct pathogenic role for anti-Yo in the present case. Previous studies have shown that patients with Ma2-Abs can be associated with cerebellar syndrome (3). Meanwhile, anti-Yo antibodies may create a synergistic effect with Ma2-Abs.CD8^+^ T cells in peripheral mononuclear cells from patients with anti-Yo antibodies have shown cytotoxic activity against neurons expressing Yo antigens (12). In patients with anti-Yo antibodies, antigenic peptides can be presented on antigen-presenting cells to stimulate cognate CD8^+^ T cells, which then enter the central nervous system and directly damage relevant neurons. Cytotoxic CD8^+^ T lymphocytes are implicated in the injury of hypocretin neurons in anti-Ma2 antibody encephalitis (5). Further studies are needed to determine whether anti-Yo antibodies can induce symptomatic narcolepsy through T cell mechanisms.

Given that both anti-Ma2 and anti-Yo antibodies are paraneoplastic autoantibodies strongly associated with tumors, our patient was thoroughly screened for tumors, but no positive findings were observed over a 3-year disease duration. Responses to immunotherapy for narcolepsy secondary to anti-Ma2 encephalitis vary according to previous reports (4, 13). Kritikou et al. proposed favorable prognostic factors for anti-Ma2 encephalitis, including male sex, absence of anti-Ma1 antibodies, age <45 years, presence of testicular cancer, complete response to antineoplastic therapy, and limited neurological involvement (13). The clinical course appears to be more favorable in patients with Ma2. All patients showed improvement or a stable condition, while the Ma patients (either Ma1 or Ma1 and Ma2) exhibited a poorer clinical prognosis (14).​Aytac et al. ‘s study claimed that anti-Yo always led to worse outcomes (6). Although former anti-Ma2 encephalitis patients’ secondary REM sleep behavior disorder(RBD) and narcolepsy are generally thought to respond poorly to immunotherapy (13), coexistence of RBD and secondary narcolepsy was still observed in this study, which may share the same autoimmune-mediated process (13). However, our patient, with coexisting anti-Ma2 and anti-Yo antibodies, presented with cataplexy as the primary symptom, associated with RBD, no detectable tumor, yet showed an unexpectedly good response to immunotherapy at the beginning. Later, however, the patient refused to continue second-line immunotherapy, and the symptoms worsened again. Vaisvilas et al. have demonstrated that I-Ma2 patients derived meaningful functional benefit from immunotherapy, with treatment efficacy indistinguishable from that observed in PNS-Ma2. Previous studies have reported one patient with anti-Ma2-associated encephalitis presenting with isolated limbic system dysfunction. Following treatment for his localized testicular tumor, the patient received IVIg and steroid therapy, which resulted in marked clinical improvement. Nevertheless, neurological relapse occurred after discontinuation of immunotherapy, and remission was achieved again upon resumption of immunotherapy (3). Therefore, it is necessary to prompt immunomodulatory intervention in patients with I-Ma2 (8).

This case presents a rare and isolated clinical manifestation of narcolepsy associated with anti-Ma2 antibody positivity with concomitant anti-Yo positivity, with cataplexy as the only early symptom. It underscores the critical role of autoimmune mechanisms in the pathogenesis of narcolepsy associated with anti-Ma2 antibody positivity and provides new insights into the early-phase efficacy of immunotherapy for narcolepsy associated with anti-Ma2 antibody positivity.

## Limitations

This study has several limitations. First, the patient did not receive standardized immunotherapy: owing to economic constraints and the patient’s own choice, he declined repeated orexin testing, and ultimately all further immunotherapy. Consequently, the value of this case for evaluating the therapeutic efficacy and prognosis of immunotherapy in Ma2-associated narcolepsy is limited, and the favorable early response observed in this patient should be interpreted with caution. Given that responses to immunotherapy in anti-Ma2 encephalitis vary considerably across previous reports (4, 13), more clinical observations and prospective controlled studies are warranted to determine the efficacy and long-term prognostic value of immunotherapy for Ma2-associated narcolepsy. Second, the patient was seropositive for anti-Yo antibodies, but the correlation between anti-Yo antibody positivity and the narcolepsy-like manifestations remain unclear. The patient exhibited no typical cerebellar phenotype, and whether anti-Yo antibodies contribute to the pathogenesis of the narcolepsy-like syndrome or merely represent an incidental serological finding could not be established in the present case. Further studies are needed to verify the significance of anti-Yo antibodies in narcolepsy-like manifestations.

## Data Availability

The original contributions presented in the study are included in the article/supplementary material. Further inquiries can be directed to the corresponding authors.
